# The acute effects of repetitive transcranial magnetic stimulation on laminar diffusion anisotropy of neocortical gray matter

**DOI:** 10.1002/mco2.335

**Published:** 2023-08-07

**Authors:** Wenjing Zhang, Naici Liu, Youjin Zhao, Chenyang Yao, Dan Yang, Chengmin Yang, Hui Sun, Xia Wei, John A. Sweeney, Huilou Liang, Miaoqi Zhang, Qiyong Gong, Su Lui

**Affiliations:** ^1^ Department of Radiology and Functional and Molecular Imaging Key Laboratory of Sichuan Province West China Hospital of Sichuan University Chengdu China; ^2^ Huaxi MR Research Center (HMRRC) West China Hospital of Sichuan University Chengdu China; ^3^ Research Unit of Psychoradiology Chinese Academy of Medical Sciences Chengdu China; ^4^ Department of Psychiatry and Behavioral Neuroscience University of Cincinnati College of Medicine Cincinnati Ohio USA; ^5^ GE Healthcare MR Research Beijing China; ^6^ Department of Radiology West China Xiamen Hospital of Sichuan University Xiamen Fujian China

**Keywords:** diffusion tensor imaging, dorsolateral prefrontal cortex, fractional anisotropy, magnetic resonance imaging, mean diffusion, repetitive transcranial magnetic stimulation

## Abstract

Repetitive transcranial magnetic stimulation (rTMS) is increasingly used to treat neuropsychiatric disorders. Inhibitory and excitatory regimens have been both adopted but the exact mechanism of action remains unclear, and investigating their differential effects on laminar diffusion profiles of neocortex may add important evidence. Twenty healthy participants were randomly assigned to receive a low‐frequency/inhibitory or high‐frequency/excitatory rTMS targeting the left dorsolateral prefrontal cortex (DLPFC). With the brand‐new submillimeter diffusion tensor imaging of whole brain and specialized surface‐based laminar analysis, fractional anisotropy (FA) and mean diffusion (MD) profiles of cortical layers at different cortical depths were characterized before/after rTMS. Inhibitory and excitatory rTMS both showed impacts on diffusion metrics of somatosensory, limbic, and sensory regions, but different patterns of changes were observed—increased FA with inhibitory rTMS, whereas decreased FA with excitatory rTMS. More importantly, laminar analysis indicated laminar specificity of changes in somatosensory regions during different rTMS patterns—inhibitory rTMS affected the superficial layers contralateral to the DLPFC, while excitatory rTMS led to changes in the intermediate/deep layers bilateral to the DLPFC. These findings provide novel insights into acute neurobiological effects on diffusion profiles of rTMS that may add critical evidence relevant to different protocols of rTMS on neocortex.

## Graphical abstract

Whole‐brain submillimeter DTI scan and a surface‐based laminar analysis were conducted to examine the inhibitory/excitatory rTMS effects on diffusion anisotropy profiles in the neocortex. The diffusion metrics of somatosensory, limbic, and sensory regions changed in opposite directions for excitatory and inhibitory rTMS and varied in cortical depths. These findings provide novel insights into acute neurobiological effects of rTMS over prefrontal cortex.

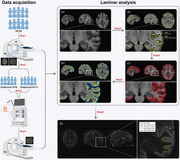

## 1 INTRODUCTION

As a noninvasive brain stimulation technique, transcranial magnetic stimulation (TMS) has emerged as an alternative interventional approach for patients with neuropsychiatric and neurological disorders who have not achieved optimal outcomes after first‐line pharmacological treatments.^1^, ^2^, ^3^ Repetitive TMS (rTMS) is known to exert therapeutic effects by inducing an electromagnetic field in the brain that results in a strong and rapidly fluctuating electrical current within the neocortex, which in turn depolarizes neurons and modulates cortical excitability at the site of stimulation.^1^, ^4^, ^5^ However, the exact neurophysiological mechanism of rTMS action remains to be fully delineated. Though it has been suggested that modulating the metaplasticity and inducing the synaptic plasticity might be one of the ways that rTMS exerts the effects,^6^ uncertainty about the full range of effects of TMS on the brain has led to some conflicting evidence on systematic application of rTMS in some psychiatric and cognitive disorders^7^, ^8^ and slowed a wider clinical use of this novel and effective therapeutic strategy.^9^, ^10^

In the past two decades, neuroimaging studies have provided insights into how TMS affects brain in anatomy, function, and chemistry. For example, magnetic resonance spectroscopy studies have demonstrated changes in γ‐aminobutyric acid concentrations in relation to TMS‐based changes in brain electrophysiology,^11^, ^12^ and positron emission tomography studies of regional cerebral blood flow/glucose metabolic rate have shown changes after long stimulation.^13^ Functional magnetic resonance imaging (fMRI) has also been used to document the acute impact of TMS on brain networks,^14^ which has shown that rTMS‐induced functional changes tend to spread distally across and within networks.^15^, ^16^ Recently, a concurrent interleaved TMS/fMRI study was conducted with state‐of‐the‐science purpose‐designed MRI head coils to delineate networks and downstream regions activated by DLPFC‐TMS, and it was found that regions of increased acute signal activation during TMS resemble a resting‐state brain network.^17^ These studies provided important progress highlighting the importance of wider network‐level consequences of TMS after site stimulation. However, the neurobiological effects of rTMS can be diverse depending on the TMS paradigms^18^, ^19^ and differential effects of low‐frequency inhibition and high‐frequency excitation across brain networks are not well characterized, especially how brain changes that occur distal to the site of TMS administration, and the laminar distribution of the effects, remain to be fully understood.

Dual‐site TMS studies have demonstrated conditioning effects in cortico–cortical pathways when stimulating two regions,^13^ and changes in such pathways may be related to modifications of microstructural properties including neurite density or myelination of fibers in gray matter,^20^, ^21^ or osmotic changes in axons due to altered cell firing rates or in extracellular water content related to perivascular changes, which can be measured with diffusion MRI.^22^ Therefore, investigating structural changes across brain gray matter after acute TMS that can be evaluated by diffusion tensor imaging (DTI), which assesses the directional diffusion of water along axon tracts might provide novel information relevant to the widely distributed effects of rTMS on the neocortex.^23^ Especially, in clinical psychiatry, the dorsolateral prefrontal cortex (DLPFC) is the most common target of TMS. Therefore, characterizing how stimulation of DLPFC causes network‐wide changes might provide critical information relevant to the full mechanistic understanding of its therapeutic efficacy.

Recent progress in image acquisition, such as the multiplexed sensitivity‐encoding (MUSE) technique,^24^ adopts a multishot scan strategy to improve image resolution and reduce distortion and blurring artifacts simultaneously. This technique has made it possible to collect high‐resolution whole‐brain DTI data within half an hour to evaluate coherent patterns of diffusion anisotropy in the gray matter at the submillimeter level. In combination with high‐resolution neuroimaging, surface‐based analysis has helped to identify the architectonic domains of diffusion orientation in the human cortex.^21^ These advances provide a useful tool for characterizing TMS‐related brain changes at the laminar level in a more manageable scan duration period, which allows for investigating rTMS effects across superficial, intermediate, and deep cell layers, which have different input, output, and cytoarchitectonic features, as are studies examining changes across infragranular, granular, and supragranular layers.^25^ When comparing “excitatory” and “inhibitory” stimulation, to the degree that differences in brain effects of these two rTMS approaches are due to a change in cell firing rates, then inverse effects of excitatory and inhibitory rTMS would be predicted, and then demonstrating such opposite effects would add confidence to data interpretation regarding changes in MRI features being related to changes in neuronal excitation during the TMS.

With these considerations in mind, a cohort of healthy participants was recruited and randomly assigned to either receiving excitatory or inhibitory rTMS, with interleaved MUSE‐DTI data collection to obtain whole‐brain diffusion data at high resolution (0.9 mm isotropic acquisition). Then, a surface‐based laminar analysis was performed to enable sampling of DTI signals within cortical layers as in previous work^21^ in order to quantify and compare the acute changes of diffusion anisotropy architecture related to rTMS excitation and inhibition. The “acute” here refers to the immediate MUSE‐DTI data collection after the rTMS stimulation which could offer a timely readout of “acute” rTMS effect in contrast to clinically observed effects that usually occur weeks after rTMS.^26^ We found that rTMS‐induced changes in diffusion anisotropy of neocortical gray matter varied in their depth across specific regions and were in opposite directions for excitatory and inhibitory rTMS. This characterization of the spatial characteristics of TMS‐induced brain changes at the laminar and regional level in water diffusion profiles may help clarify the differential effects of rTMS paradigms on the neocortex that future applications might refer to for specific treatment purpose.

## 2 RESULTS

### 2.1 Demographic characteristics

**FIGURE 1:**
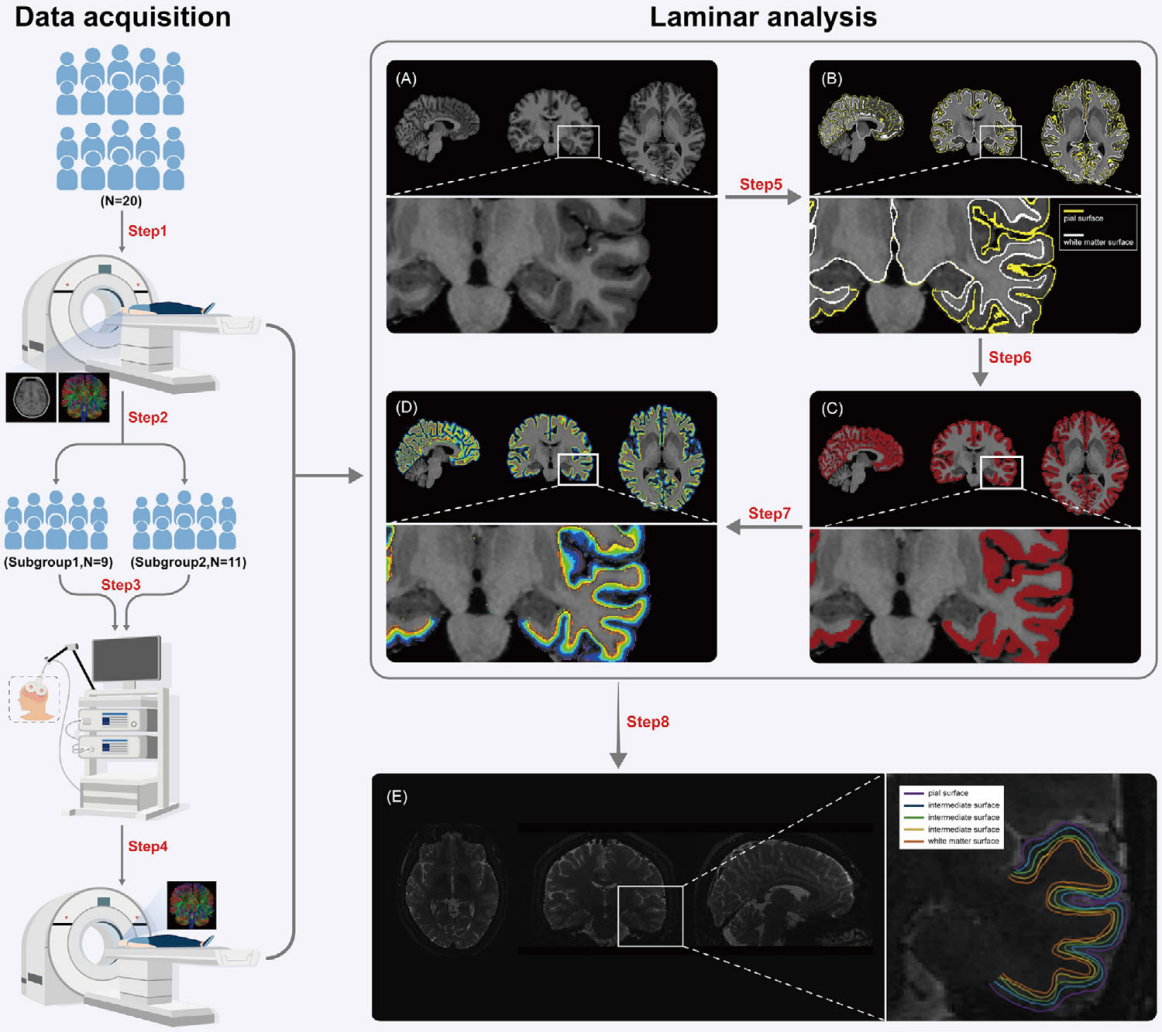
Flowchart of data acquisition and laminar analysis. During the data acquisition, participants were scanned with high‐resolution MRI at baseline (Step 1) and randomly divided into two subgroups (Step 2). Participants in subgroup 1 and subgroup 2 each received inhibitory or excitatory rTMS targeting the left DLPFC after the baseline MR scan (Step 3) and were scanned again immediately after the rTMS session (Step 4). After data collection, laminar analysis was then performed. The preprocessed high‐resolution T1‐weighted images were used to generate the surface of pial and white matter (Step 5, the reconstructed pial surface is marked with yellow line, while the white matter surface is white line). The layer segmentation based on the surfaces above was conducted and the reconstruction of the segmentation was superimposed as red ribbon (Step 6). Equal‐distance method was adopted and 11 layers based on 10 intermediate surfaces were evenly generated and superimposed as ribbons with different colors (Step 7). The DTI data were registered with the laminar information built with high‐resolution T1‐weighted images (Step 8, each line overlaid on the DTI axial slice represented a specific layer and 5 layers were shown here as sketch map). MRI‐magnetic resonance imaging, DTI‐diffusion tensor imaging, rTMS‐repetitive transcranial magnetic stimulation, DLPFC‐dorsolateral prefrontal cortex.

In order to measure the acute changes of diffusion anisotropy architecture related to different rTMS patterns, 20 healthy participants were randomly assigned to receiving a single session of low‐frequency inhibitory rTMS (subgroup 1, *n* = 9, seven females, age: 23.56 ± 2.41 years, education: 16.89 ± 1.54 years) or high‐frequency excitatory rTMS (subgroup 2, *n* = 11, nine females, age: 25.82 ± 2.56 years, education: 19.18 ± 2.04 years), both targeting left DLPFC. A within‐subject cross‐over design was not used to avoid the potential effects of prior stimulation on response to the second stimulation. Participants were scanned immediately before and after the rTMS session, and laminar analysis was conducted with the collected MRI data. The flowchart of study procedures is presented in Figure 1. There were no significant differences in age or sex distribution between groups, while the latter had a significantly higher educational level than the former (Table 1).

**TABLE 1:** Demographics of healthy participants.

Demographic variables	Participants receiving inhibitory rTMS (*N* = 9)		Participants receiving excitatory rTMS (*N* = 11)			
	Mean	SD	Mean	SD	*t*	*p* Value
Age (year)	23.56	2.41	25.82	2.56	1.97	0.065
Education (year)	16.89	1.54	19.18	2.04	2.78	0.012

Sex	*N* (%)		*χ*	*p* Value
Male	2 (22%)	2 (18%)	0.51	0.822
Female	7 (78%)	9 (82%)		

Abbreviations: rTMS, repetitive transcranial magnetic stimulation; SD, standard deviation.

### 2.2 Effects of rTMS inhibition on diffusion metrics in laminar cortex

**FIGURE 2:**
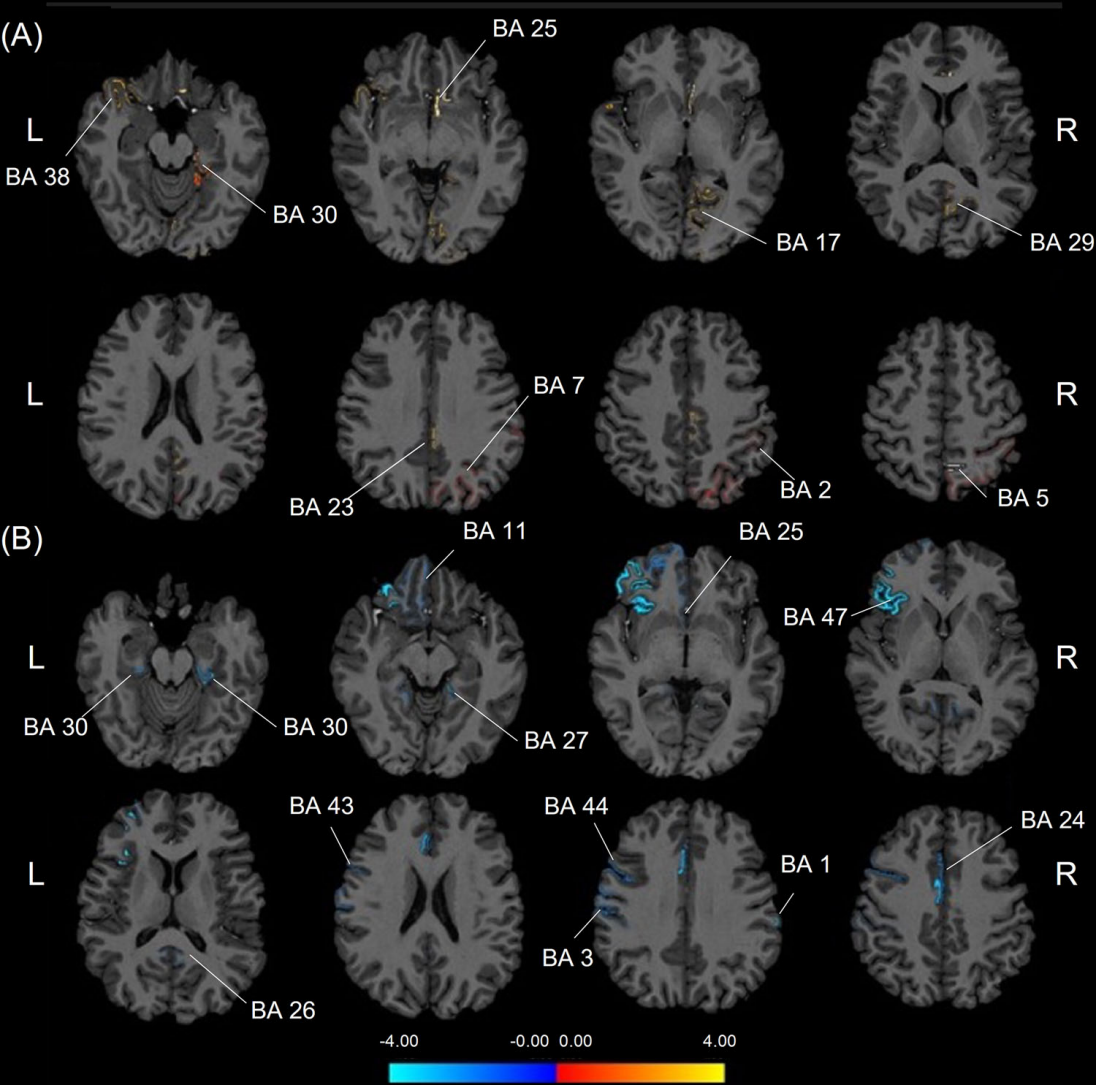
The cortical layers with significant alternations of fractional anisotropy values after (A) inhibitory or (B) excitatory rTMS. The increased fractional anisotropy values are indicated by red/warm color and the decreased fractional anisotropy values are indicated by blue/cold color. BA, Brodmann's area; L, left hemisphere; R, right hemisphere; rTMS, repetitive transcranial magnetic stimulation.

**FIGURE 3:**
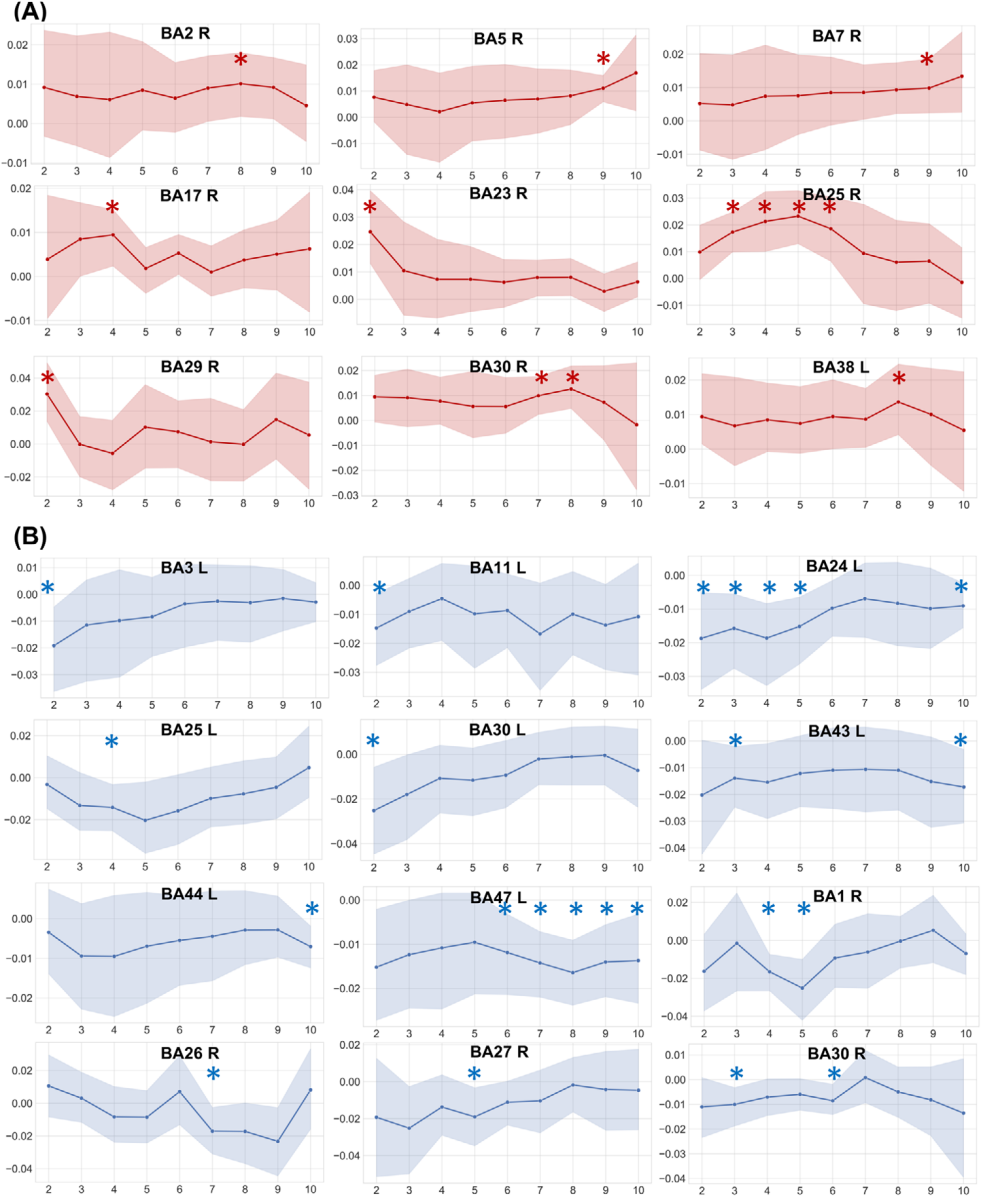
The chart of altered fractional anisotropy (FA) values in multiple cortical layers after (A) inhibitory or (B) excitatory rTMS. The *X*‐axis is layer and the *Y*‐axis is △FA values. The △FA values = FA values after simulation—FA values at baseline. The increased FA is indicated by the red/warm color, while the decreased FA is the blue/cold color. * indicates the specific layer(s) with significant alternations before and after rTMS. The band indicates the 95% confidence interval of △FA values. BA, Brodmann's area: L, left hemisphere; R, right hemisphere.

To characterize the acute effects of inhibitory stimulation on diffused properties of laminar cortex, a paired‐sample *t*‐test was performed to obtain the laminar fractional anisotropy (FA) and mean diffusivity (MD) changes in cortical gray matter before and after the inhibitory rTMS. After rTMS inhibition, participants showed increased FA values mainly in primary/secondary somatosensory regions (right primary somatosensory cortex [Brodmann's area (BA) 2], right somatosensory association cortex [BA 5], and right visuo‐motor coordination regions [BA 7]). Increased FA was also seen in the cingulate cortex (right ventral posterior cingulate cortex [BA 23], right isthmus of the cingulate cortex [BA 29 and BA 30], and right subgenual cingulate cortex [BA 25]), sensory areas including right primary visual cortex (BA 17), and regions involved in affect modulation including the left temporal pole (BA 38). Notably, the FA changes of somatosensory regions were observed in more superficial layers within gray matter, while those in extended limbic circuitry and sensory regions were deeper. The specific layers of gray matter regions with FA increase are shown in Table 2, Figures 2A and 3A. No FA decrease was observed in any region after rTMS inhibition.

**TABLE 2:** The alternations of fractional anisotropy values after rTMS inhibition.

			Mean (SD)			*p* Value	
Gray matter regions	Laminar layers	Estimated histological layers	Baseline	After rTMS inhibition	*t*	Uncorrected	FDR corrected
Primary somatosensory cortex. R (BA2)	8	III	0.217 (0.010)	0.227 (0.014)	2.34	0.048	0.239
Somatosensory association cortex. R (BA5)	9	II	0.241 (0.012)	0.252 (0.013)	3.96	0.004	0.037^a^
Visuo‐motor coordination. R (BA7)	9	I–II	0.224 (0.018)	0.233 (0.019)	2.36	0.046	0.204
Primary visual cortex. R (BA17)	4	IV	0.276 (0.025)	0.285 (0.024)	2.73	0.026	0.231
Ventral posterior cingulate cortex. R (BA23)	2	VI	0.328 (0.017)	0.353 (0.031)	3.26	0.012	0.105
Subgenual area. R (BA25)	3	III–VI	0.350 (0.026)	0.368 (0.022)	4.32	0.003	0.011^a^
	4		0.345 (0.024)	0.366 (0.024)	3.44	0.009	0.027^a^
	5		0.337 (0.021)	0.360 (0.021)	4.49	0.002	0.011^a^
	6		0.343 (0.026)	0.361 (0.027)	2.92	0.019	0.044^a^
Retrosplenial cingulate cortex. R (BA29)	2	VI	0.402 (0.052)	0.432 (0.057)	3.23	0.012	0.109
Part of cingulate cortex. R (BA30)	7	III	0.304 (0.021)	0.314 (0.025)	2.45	0.040	0.181
	8		0.298 (0.026)	0.311 (0.029)	2.66	0.029	0.181
Temporal pole. L (BA38)	8	III	0.272 (0.012)	0.285 (0.024)	2.51	0.036	0.284

Abbreviations: L, left hemisphere; R, right hemisphere; BA, Brodmann's area; rTMS, repetitive transcranial magnetic stimulation; SD, standard deviation; FDR, false discovery rate.

^a^ Indicates FDR corrected *p* value < 0.05.

With regard to changes in regional MD laminar profiles, primary/secondary somatosensory regions (right primary somatosensory cortex [BA 1], right visuo‐motor coordination region [BA 7], left somatosensory association cortex [BA 5]), bilateral cingulate cortex (right BA 24 and left BA 29), and sensory regions (bilateral temporal association cortex [right BA 22 and left BA 38], bilateral auditory cortex [right BA 41 and bilateral BA 42], and right secondary visual cortex [BA 18]) showed decreased MD values, which were primarily in middle and deep gray matter (Table S1). In contrast, laminar profiles in multiple frontal lobe regions showed increased MD values, including bilateral DLPFC (right BA 9 and left BA 46), bilateral anterior prefrontal cortex (bilateral BA 10), and left lateral and medial premotor cortex (BA 6) and regions of left BA 8. Most of these changes were observed at superficial to middle layers within gray matter (Table S2).

These results indicated that cortical regions with significant changes in FA and MD were mainly located in somatosensory, limbic, and sensory regions after inhibitory rTMS. Specifically, cortical regions only showed increased FA after inhibitory rTMS, and the conditioning effects induced by inhibitory rTMS were observed in the superficial layers of somatosensory regions contralateral to the DLPFC.

### 2.3 Effects of rTMS excitation on diffusion metrics in laminar cortex

The same analysis was also adopted to characterize the acute effects of excitatory stimulation. After excitatory rTMS, participants showed decreased FA values in widespread regions, including bilateral primary somatosensory cortex (right BA 1 and left BA 3), left frontal regions (left orbitofrontal area [BA 11], left pars opercularis [BA 44], and left pars orbitalis [BA 47]), cingulate regions (left ventral anterior cingulate cortex [BA 24], bilateral isthmus of cingulate gyrus [bilateral BA 30], a left subgenual area [BA 25], and a right ectosplenial area [BA 26]), and some sensory regions (right piriform cortex [BA 27] and left primary gustatory cortex [BA 43]). FA changes in inferior frontal regions were more superficial within gray matter, while those in cingulate and sensory regions were deeper. Laminar effects in gray matter regions are shown in Table 3, Figures 2B and 3B. No FA increase was observed in any region after rTMS excitation.

**TABLE 3:** The alternations of fractional anisotropy values after rTMS excitation.

			Mean (SD)			*p* Value	
Gray matter regions	Laminar layers	Estimated histological layers	Baseline	After rTMS excitation	*t*	Uncorrected	FDR corrected
Primary somatosensory cortex. R (BA 1)	4	V	0.254 (0.041)	0.238 (0.039)	−3.10	0.011	0.075
	5		0.262 (0.067)	0.237 (0.062)	−2.87	0.017	0.075
Primary somatosensory cortex. L (BA 3)	2	VI	0.343 (0.031)	0.324 (0.020)	−2.31	0.043	0.389
Orbitofrontal area. L (BA 11)	2	VI	0.371 (0.045)	0.356 (0.034)	−2.27	0.047	0.377
Ventral anterior cingulate cortex. L (BA 24)	2	I, III–VI	0.298 (0.023)	0.279 (0.013)	−2.41	0.037	0.066
	3		0.267 (0.022)	0.251 (0.016)	−2.62	0.026	0.066
	4		0.257 (0.024)	0.239 (0.012)	−2.84	0.018	0.066
	5		0.250 (0.016)	0.235 (0.013)	−2.83	0.018	0.066
	10		0.228 (0.021)	0.219 (0.016)	−2.42	0.036	0.066
Subgenual area. L (BA 25)	4	V	0.364 (0.036)	0.350 (0.032)	−2.36	0.040	0.226
Ectosplenial area. R (BA 26)	7	III	0.295 (0.015)	0.278 (0.031)	−2.28	0.046	0.309
Piriform cortex. R (BA 27)	5	II	0.370 (0.038)	0.351 (0.038)	−2.27	0.047	0.321
Part of cingulate cortex. L (BA 30)	2	VI	0.356 (0.043)	0.330 (0.037)	−2.34	0.042	0.375
Part of cingulate cortex. R (BA 30)	3	III, VI	0.313 (0.028)	0.303 (0.021)	−2.40	0.038	0.169
	6		0.307 (0.021)	0.299 (0.021)	−2.64	0.025	0.169
Primary gustatory cortex. L (BA 43)	3	I, V	0.273 (0.022)	0.259 (0.020)	−2.25	0.048	0.187
	10		0.238 (0.032)	0.221 (0.037)	−2.35	0.041	0.187
Pars opercularis. L (BA 44)	10	I	0.222 (0.026)	0.215 (0.022)	−2.39	0.038	0.343
Pars orbitalis. L (BA 47)	6	I–III	0.272 (0.028)	0.260 (0.026)	−2.43	0.035	0.063
	7		0.273 (0.030)	0.258 (0.028)	−3.55	0.005	0.024^a^
	8		0.271 (0.028)	0.255 (0.026)	−4.25	0.002	0.015^a^
	9		0.258 (0.027)	0.244 (0.025)	−3.21	0.009	0.028^a^
	10		0.258 (0.033)	0.244 (0.030)	−2.52	0.030	0.063

Abbreviations: L, left hemisphere; R, right hemisphere; BA, Brodmann's area; rTMS, repetitive transcranial magnetic stimulation; SD, standard deviation; FDR, false discovery rate.

^a^ Indicates FDR corrected *p* value < 0.05.

With regard to effects on MD measurements, right orbitofrontal area (BA 11), right ectosplenial cortex (BA 26), left cingulate cortex (BA 30), and left primary visual cortex (BA 17) showed decreased MD values distributed across gray matter layers (Table S3). In contrast, laminar profiles in right cingulate cortex (BA 30), right dorsal entorhinal cortex (BA 34), and right inferior temporal gyrus (BA 20) showed increased MD values at superficial to middle layers within gray matter (Table S4).

These results indicated that cortical regions with significant changes in FA and MD were also located in somatosensory, limbic, and sensory regions after excitatory rTMS. Nevertheless, cortical regions only showed decreased FA after excitatory rTMS, and the conditioning effects induced by excitatory rTMS led to changes in the intermediate/deep layers of somatosensory regions in both hemispheres.

### 2.4 Difference in diffusion metrics after rTMS excitation and inhibition

To compare the effects of excitation and inhibition stimulation, differences (△values = values after simulation − values at baseline) in FA and MD values after rTMS stimulation were compared across the two study groups using two‐sample *t*‐tests. In a direct comparison of responses to excitatory and inhibitory stimulation, participants after rTMS inhibition showed larger increases in FA in bilateral primary somatosensory cortex (right BA 1 and left BA 3), bilateral cingulate regions (right BA 23, bilateral BA 24, bilateral BA 30 and bilateral BA 32), right subgenual area (BA25), left temporal pole (BA 38), and left interior frontal gyrus (BA 47) than participants who had excitatory rTMS.

Inhibitory rTMS was associated with larger increases in MD in right primary somatosensory cortex (BA 3), right prefrontal cortex (BA 10), right orbitofrontal area (BA 11), left primary visual cortex (BA 17), left fusiform gyrus (BA 37), left temporal pole (BA 38) and cingulate regions (right BA 26 and left BA 30), and larger decreases in MD in right primary somatosensory cortex (BA 1) and left temporal pole (BA 38) relative to participants who had excitatory rTMS. Detailed intergroup comparison of the laminar profiles of effects of excitatory and inhibitory rTMS are presented in Tables S5 and S6.

These findings showed that participants after rTMS inhibition had larger increases in FA and larger alternations in MD in several regions than participants who had excitatory rTMS by directly comparing the changes in FA and MD values before and after each pattern of rTMS.

## 3 DISCUSSION

In this study, to contrast the acute effects of rTMS excitation and inhibition on neocortical gray matter, we performed a laminar analysis with high‐resolution whole‐brain diffusion data. Several significant findings were demonstrated. First, brain regions with diffusion anisotropy changes were widely distributed in regions distal from the stimulation site, mainly in somatosensory regions, limbic regions, and some sensory regions, after both TMS excitation and inhibition. Second, regions with changes in diffusion measures after inhibitory rTMS all showed increased FA values, and most of these effects were contralateral to the stimulating site. In contrast, after excitatory rTMS, regions with altered diffusion measures all showed decreased FA values and the affected regions were most often ipsilateral to the stimulation site (rTMS excitation also decreased FA values of ipsilateral frontal regions). Third, the early post‐TMS cortico–cortical pathways underlying conditioning effects appear to involve the superficial layers of somatosensory regions contralateral to the stimulating site of inhibition, and the intermediate/deep layers of these regions bilaterally near the stimulating site of excitation. Effects were also seen across all layers in some sensory regions, most notably in the middle layers within gray matter.

The first important finding of our study is that both inhibitory and excitatory rTMS consistently and mainly stimulate diffusion profile changes in somatosensory regions, limbic regions, and some sensory regions. This suggests that the changes in diffusion anisotropy induced by rTMS are widespread, well beyond the site of stimulation, which highlights how focal rTMS can have broad‐ranging impact on brain regions/networks involved in emotion and cognitive processing.^27^ The activated regions via our rTMS paradigms and diffusion measures were quite similar to those recently measured with functional MRI,^17^ in which brain regions in DLPFC, inferior parietal lobe, and anterior cingulate cortex all have been observed with increased acute signal activation during TMS.^17^ More importantly, in a study that investigated treatment effects of rTMS with DLPFC targeting in depression, these regions have also been observed with alterations after a seed‐based connectivity analysis.^28^ Notably, somatosensory regions are related to proprioception,^29^ while limbic regions including cingulate cortex usually are in charge of receiving inputs or external stimulations from other brain regions and involved in complicated and interconnected cognitive and emotional processing.^30^ While the TMS has been suggested to act through a top‐down mechanism,^27^ it could be inferred that some specific neural circuit is evoked after DLPFC stimulation, in which somatosensory regions percept the stimulations whereas the limbic regions process them. Further studies are warranted for the precise characterization of the abovementioned physiologic processes in order to understand their potential contribution to treating cognitive and emotional processing impairments.

The second important finding is that the specific changes of FA after TMS excitation and inhibition were in opposite directions. Several mechanisms might underpin these changes. First, FA values in part reflect myelination of axons,^31^ which in the cortex are aligned perpendicular to the cortical surface.^32^, ^33^, ^34^ Collateral branches of axons and dendrites of neurons branch in less organized ways, but the primary large axons are organized directionally to provide a directional diffusion signal.^35^ In previous studies of multiple sclerosis, the loss of these axons and their myelination leads to a decrease of FA values in the neocortex, while FA values in some regions can be increased due to the concomitant loss of axonal branching.^34^ While these findings highlight the biological significance of diffusion imaging in gray matter, such changes seem unlikely to account for the observed diffusion changes after acute rTMS. Aside from rapid changes in myelination, regional changes in neuronal membrane permeability or water volume content in pyramidal cell axons following marked increases (excitatory rTMS) and decreases (inhibitory rTMS) in their cell firing rates during TMS might also contribute to the acute regional diffusion changes. High‐frequency and low‐frequency TMS treatment depolarize different kinds of neurons^18^ leading to changes in neurophysiology outside the normal range of cell firing rates, which could cause acute neuronal swelling via mechanisms that would differentially impact aggregate FA measurements.^36^, ^37^, ^38^

Another mechanism that might contribute to the difference of FA changes after excitatory and inhibitory rTMS is the differential neurotransmitter concentration across cortical layers. A recent examination of the glutamate signal changes in response to theta‐burst stimulation indicated a pattern of decreases in the ipsilateral hemisphere.^11^ This might underpin our observed changes that after rTMS excitation, the FA values of ipsilateral regions were more likely to change while after rTMS inhibition, the FA values of contralateral were more likely to change. The distinct effects of rTMS were attributed to those fibers within the cortical region stimulated which projected ortho‐ or antidromically to distant structures.^39^, ^40^ However, more work is need to clarify the fundamental neurobiological cause of such effects, and their duration.

The third important finding is that we observed increased FA in superficial layers of somatosensory regions after inhibition but decreased FA in deeper layers after excitation, while the limbic and sensory regions showed no laminar specificity of FA changes. Such laminar differences suggest that different cortical layers in somatosensory regions react differently to rTMS frequency, while the cause of such effects and potential clinical implications remains to be determined. Notably, though the segmented lamina does not correspond directly to cortical layers which are cytoarchitecturally defined and vary their spacing across neocortex, our analysis does suggest that superficial and deep layers in these regions respond quite differently to excitatory and inhibitory rTMS.

In addition to diffusion metric changes in the brain regions as noted above, there were also FA or MD alterations in frontal cortex, mainly in intermediate and superficial layers. The FA values were found to decrease in several left frontal regions after rTMS excitation, and increased MD was demonstrated mainly in left (ipsilateral) frontal regions after rTMS inhibition. Such findings suggested that after stimulating left DLPFC, other frontal regions can also be simulated as they are interconnected part of intrinsic functional brain networks of frontal cortex.^15^ The MD changes are noteworthy, as MD measures are sensitive to cellularity and edema,^41^ so that MD in frontal regions after inhibition might manifest as a result of subtle brain edema. While our findings demonstrate that focal rTMS can have wide ranging effects on the anatomy of neocortical gray matter, putatively by changing cell firing rates to extraphysiological levels, whether these acute changes represent clinically relevant adverse effects and whether they persist beyond the immediate post rTMS period remain to be determined.

Some limitations are noteworthy regarding the current study. First, we did not include a sham control group to rule out the possibility that the results might be confounded by nonspecific effects of rTMS. Our observations of opposite changes after excitatory and inhibitory rTMS reduce concerns about such artifacts to a degree. Second, we only examined the rTMS effects after single stimulation in left DLPFC. While this is a common stimulation site in clinical practice, effects following rTMS to other brain regions remain to be determined. Third, in our laminar analysis, the layers do vary as a percent of depth in neocortex, they do not directly correspond with histologically defined cortical layers I–VI. Examination of effects in cytoarchitecturally defined layers and clarification of causes of diffusion changes might best be obtained from future preclinical research with animal models. Fourth, the sample size of current analysis is not large and the sex distributions were not balanced (there were only two male participants in each group). Therefore, our findings should be considered with caution. Replication of our findings in larger studies is needed in the future. Fifth, though we characterized the rTMS in each participant group, the difference in education level might also confound the findings, which should be taken into consideration in future work especially when two participant groups are directly compared. Finally, studies from the clinical perspective need to confirm that our findings do not reflect clinically meaningful adverse effects on motor, sensory, affective or cognitive function, and the persistence of the findings needs to be examined with longer term follow‐up studies.

## 4 METHODS AND MATERIALS

### 4.1 Participants

This study was approved by the Research Ethics Committee of West China Hospital and performed in accordance with the Declaration of Helsinki. Written informed consent was obtained from all study participants. In this study, twenty healthy participants were recruited through poster advertisements from the nearby Chengdu community. All participants were right‐handed and of Chinese Han ancestry. The age range for included participants was 20−31 years. Participants were excluded if they had: (1) contraindications to MRI scanning (e.g., claustrophobia or braces), (2) a history of substance abuse or dependence, (3) a positive pregnancy test, (4) a history of major systemic disease, known psychiatric/neurological illness, or an episode of loss of consciousness >10 min, and (5) a history of serious mental illness in their first‐degree relatives. Notably, a large cohort of healthy participants for rTMS recording had been established elsewhere before.^42^

### 4.2 Repetitive TMS

Repetitive TMS was delivered using a M‐100 Ultimate magnetic stimulator (Shenzhen Yingchi Technology Co. Ltd) and a 70 mm figure 8 coil that stimulated a 2 cm^2^ area that extended 2 cm into the cortex.^43^ The coil was held tangentially to the scalp with the handle pointing back and away from the midline at 45°. Brodmann Area 9 (BA 9; MNI coordinates: *x* = −23.1, *y* = 37.4, *z* = 43.0) was used as the cortical landmark of left DLPFC.^44^ The neuro‐navigation system (QuickVision; Shenzhen Yingchi Technology Co. Ltd) was used for precise localization,^45^ with coordinates provided by incorporating pre‐stimulation high‐resolution brain anatomical scans into the navigator. The resting motor threshold (RMT) was measured before the subsequent rTMS stimulation by stimulating the M1 region of the left cerebral hemisphere in a single stimulation mode and determined by involuntary contraction of the fingers to provide the reference baseline.^46^, ^47^ High‐frequency rTMS stimulation (≥5 HZ) has been associated predominantly with excitatory effects while low‐frequency rTMS (≤5 HZ) stimulation has predominantly inhibitory effects.^48^, ^49^ In this study, inhibitory rTMS was applied at 1 Hz with an intensity of 100% of RMT, and with 120 trains of 5 s duration with 5 s between trains. Excitatory rTMS was applied at 15 Hz with an intensity of 80% of RMT, with 100 trains of 1 s duration and 11 s between trains.

### 4.3 Data acquisition

All MRI data were collected on a 3.0T MRI scanner (Signa Premier; GE Healthcare) with a 48‐channel head coil. A MUSE technique was used to acquire submillimeter whole‐brain isotropic DTI data with parameters as follows: *b* values = 0, 800 s/mm^2^, number of directions = 13, number of shots = 4, repetition time (TR) = 22,531 ms, echo time (TE) = 83.1 ms, flip angle = 90°, number of excitations = 1, fat suppression technique was turned on, in‐plane resolution = 0.9 × 0.9 mm^2^ and a 256 × 256 matrix over a field of view (FOV) of 230 × 230 mm^2^ resulting in 150 axial slices of 0.9 mm thickness covering the whole brain without a gap. The nominal scan time was about 23 min. During the scanning, the distortion correction feature provided on the scanner was turned on to reduce residual distortions of MUSE.^50^ High‐resolution T1‐weighted images (T1WI) were acquired with a Magnetization Prepared Rapid Gradient Echo (MPRAGE) sequence: TR/TE = 9.6/2.32 ms, flip angle = 8°. A FOV of 240 × 240 mm^2^ was used with an acquisition matrix comprising 256 readings of 128 phase‐encoding steps, producing 168 contiguous 1.0 mm axial slices. The final matrix of T1‐weighted images was automatically interpolated in plane to 512 × 512, yielding an in‐plane resolution of 0.47 × 0.47 mm^2^.

During the MRI scan, earplugs and headphones were provided to block background noise, and foam padding around the head minimized head motion. Obtained brain images including both T1WI and DTI were visually inspected by two experienced neuroradiologists, and no scan artifacts or gross brain abnormalities were observed in any participant.

### 4.4 Data processing and laminar depth analysis

DTI data preprocessing, including denoising^51^ and eddy current correction,^52^ was performed with MRtrix3 (https://www.mrtrix.org/) and FSL (https://fsl.fmrib.ox.ac.uk/fsl/). The preprocessed DTI images were used to generate FA maps with MRtrix3.^53^ The DTI images were registered to T1‐MPRAGE scans using epi_reg provided in FSL, which incorporates white matter boundary information in the registration process.^54^, ^55^

T1‐MPRAGE images were preprocessed with FreeSurfer (version 7.1.0, http://surfer.nmr.mgh.harvard.edu/) to generate pial surface and white matter surface maps, which were transformed into volume space with AFNI and SUMA (https://afni.nimh.nih.gov/). The pial surface was defined as the boundary between cortical gray matter and cerebrospinal fluid (CSF). The white matter surface represented the boundary between white matter and cortical gray matter. Finally, the layer segmentation based on pial surface and white matter surface was conducted with LAYNII (https://layerfmri.com/2020/05/29/laynii‐setup/#more‐2378). An equal‐distance method was adopted, with 10 intermediate surfaces evenly spaced throughout the cortical depth being identified,^56^ as done previously.^21^ Finally, 11 layers were obtained, with the while matter surface being referred to as the 1st surface, the next closest surface as the 2nd surface and so on to the pial surface, the most superficial gray matter (Figure S1). Since cytoarchitectural layers cannot be defined in MRI images, so patterns of effects across defined layers could be informative about supragranular, granular, and infragranual layer of cortex. The predefined Brodmann template that defined 82 cortical regions^57^ was adopted in this study. Diffusion anisotropy profiles including FA and MD metrics were analyzed by calculating the average measures of the FA and MD metrics across voxels in each layer of each Brodmann region. This laminar subdivision segments the cortex that is ∼3 mm thick into depth regions (“layers,” each layer has about 0.3 mm thickness) that are approximately three times thinner than our 0.9 mm voxels. The first and last layers in the segmentation were discarded because of the potential confounding effects of white matter or CSF nearby.

### 4.5 Statistical analyses

To characterize the acute effects of rTMS on micro‐structural properties of cortical gray matter within each of the two study groups considered separately, we tested for laminar FA and MD changes in neocortex after excitatory and inhibitory stimulations using paired‐sample *t*‐tests. A *p* value (two‐tailed) less than 0.05 was considered to be statistically significant. In addition, *p* values of each brain region were corrected with false discovery rate (FDR) by using the code “FDR_p = mafdr(p,‘BHFDR’, true)” in Matlab to correct the effects of multiple layers. The code refers to the Benjamini–Hochberg FDR method,^58^ which ranks the p values and adjusts them based on their ranking and controls the FDR at a specified level. To contrast the effects of excitation and inhibition stimulation, changes in FA and MD values after TMS stimulation were compared across the two study groups using two‐sample *t*‐tests.

## 5 CONCLUSIONS

By characterizing the laminar profiles of cortical diffusion anisotropy induced by rTMS, a rapid change in water diffusion in neocortex especially within brain circuits that are involved in sensory systems, cognition, and emotion processing after either excitatory and inhibitory rTMS. These effects varied across regions in their depth from the cortical surface, and were in opposite directions for excitatory and inhibitory rTMS. These findings provide novel insights into the acute neurobiological effects of different protocols of rTMS administered over prefrontal cortex.

## AUTHOR CONTRIBUTION

Drs. Wenjing Zhang, Qiyong Gong, and Su Lui carefully designed the research. Drs. Youjin Zhao and Chenyang Yao performed rTMS to participants. Drs. Huilou Liang and Miaoqi Zhang adjusted MRI sequences. Ms. Dan Yang, Drs. Chengmin Yang, Hui Sun, and Xia Wei collected MRI and clinical data. Dr. Naici Liu preprocessed the imaging and clinical data, conducted the surface‐based laminar analysis and statistical analyses, and produced tables and figures. Drs. Naici Liu and Wenjing Zhang wrote the first draft of this manuscript, while Dr. John A. Sweeney made critical revision of the manuscript. Dr. Wenjing Zhang revised the manuscript to form the final version. All authors gave final approval of the version to be published.

## CONFLICT OF INTEREST STATEMENT

Drs. Wenjing Zhang and John A. Sweeney consult to VeraSci. Drs. Huilou Liang and Miaoqi Zhang are employees of GE Healthcare, MR Research, Beijing, China. The company has no role in designing and performing the study or analyzing and interpreting the data. The remaining authors have no competing interests or financial support to disclose.

## ETHICS STATEMENT

All procedures involving human participants were approved by the Research Ethics Committee of West China Hospital of Sichuan University (reference NO. 1016) and performed in accordance with the Declaration of Helsinki. All participants provided written informed consent before study participation.

## Supporting information

Supporting Information

## Data Availability

West China Hospital of Sichuan University has an institutional commitment to data sharing. To get access to the data and comply with the terms of our research ethics committee approval, an application to the corresponding author will be required, specifying the geographical extent of sharing.
