# Supplementary material for: The acute effects of repetitive transcranial magnetic stimulation on laminar diffusion anisotropy of neocortical gray matter
Source: MedComm (2020). 2023 Aug 7;4(4):e335. doi: 10.1002/mco2.335 (PMC10407029; doi:10.1002/mco2.335)
Supplement: Supplementary file 1 — Supporting Information [file MCO2-4-e335-s001.docx]

**The acute effects of repetitive transcranial magnetic stimulation on laminar diffusion anisotropy of neocortical gray matter**

Wenjing Zhang^1, 2, 3, #^, Naici Liu^1, 2, 3, #^, Youjin Zhao^1, 2, 3^, Chenyang Yao^1, 2, 3^, Dan Yang^1^, Chengmin Yang^1, 2, 3^, Hui Sun^1, 2, 3^, Xia Wei^1, 2, 3^, John A. Sweeney^2, 4^, Huilou Liang^5^, Miaoqi Zhang^5^, Qiyong Gong^1, 2, 3, 6^, Su Lui^1, 2, 3, *^

1. Department of Radiology, and Functional and Molecular Imaging Key Laboratory of Sichuan Province, West China Hospital of Sichuan University, Chengdu, China
2. Huaxi MR Research Center (HMRRC), West China Hospital of Sichuan University, Chengdu, China
3. Research Unit of Psychoradiology, Chinese Academy of Medical Sciences, Chengdu, China.
4. Department of Psychiatry and Behavioral Neuroscience, University of Cincinnati College of Medicine, Cincinnati, OH, USA
5. GE Healthcare, MR Research, Beijing, China.
6. Department of Radiology, West China Xiamen Hospital of Sichuan University, Xiamen, Fujian, China

^#^ These authors contributed equally to this work and shared first authorship.

**^*^ Correspondence:**

Dr. Su Lui, No. 37 Guoxue Xiang, Chengdu 610041, China.

Email: lusuwcums@tom.com.

Table S1. The decreased mean diffusion values after rTMS inhibition.

| **Gray matter regions** | **Laminar layers** | **Baseline** | **After rTMS**  **inhibition** | **t** | **p values** | |
| --- | --- | --- | --- | --- | --- | --- |
|  |  | **Mean (SD)** | |  | **uncorrected** | **FDR corrected** |
| Primary Somatosensory Cortex1 R  (BA 1) | 3 | 0.00082 (0.00014) | 0.00077 (0.00010) | -2.72 | 0.026 | 0.133 |
|  | 5 | 0.00084 (0.00014) | 0.00080 (0.00012) | -2.64 | 0.030 | 0.133 |
| Somatosensory Association Cortex L  (BA 5) | 5 | 0.00073 (0.00003) | 0.00072 (0.00003) | -2.39 | 0.044 | 0.265 |
| Visuo-Motor Coordination R  (BA 7) | 2 | 0.00080 (0.00003) | 0.00079 (0.00002) | -3.14 | 0.014 | 0.124 |
| Secondary visual cortex (V2) R  (BA 18) | 5 | 0.00075 (0.00001) | 0.00074 (0.00002) | -2.52 | 0.036 | 0.203 |
|  | 6 | 0.00077 (0.00002) | 0.00076 (0.00002) | -2.37 | 0.045 | 0.203 |
| Superior temporal gyrus R  (BA 22) | 2 | 0.00079 (0.00003) | 0.00077 (0.00004) | -2.80 | 0.023 | 0.208 |
| Ventral anterior cingulate cortex R  (BA 24) | 2 | 0.00083 (0.00004) | 0.00081 (0.00004) | -2.34 | 0.048 | 0.391 |
| Subgenual area R  (BA 25) | 6 | 0.00076 (0.00006) | 0.00074 (0.00005) | -2.75 | 0.025 | 0.186 |
| Retrosplenial cingulate cortex L  (BA 29) | 3 | 0.00077 (0.00006) | 0.00074 (0.00006) | -2.39 | 0.044 | 0.216 |
|  | 4 | 0.00080 (0.00007) | 0.00076 (0.00006) | -2.33 | 0.048 | 0.216 |
| Temporal pole L  (BA 38) | 4 | 0.00082 (0.00002) | 0.00081 (0.00004) | -2.36 | 0.046 | 0.285 |
| Auditory cortex1 R  (BA 41) | 2 | 0.00076 (0.00003) | 0.00073 (0.00003) | -2.73 | 0.026 | 0.231 |
| Auditory cortex2 L  (BA 42) | 3 | 0.00077 (0.00004) | 0.00076 (0.00003) | -3.08 | 0.015 | 0.136 |
| Auditory cortex2 R  (BA 42) | 2 | 0.00084 (0.00003) | 0.00081 (0.00002) | -2.67 | 0.029 | 0.257 |

Abbreviations: L-left hemisphere, R-right hemisphere, BA-Brodmann’s area, rTMS-repetitive transcranial magnetic stimulation, SD-Standard Deviation, FDR-false discovery rate.

Table S2. The increased mean diffusion values after rTMS inhibition.

| **Gray matter regions** | **Laminar layers** | **Baseline** | **After rTMS inhibition** | **t** | **p values** | |
| --- | --- | --- | --- | --- | --- | --- |
|  |  | **Mean (SD)** | |  | **uncorrected** | **FDR corrected** |
| Premotor cortex and Supplementary Motor Cortex L  (BA 6) | 9 | 0.00082 (0.00008) | 0.00085 (0.00007) | 2.48 | 0.038 | 0.244 |
| Frontal eye fields L  (BA 8) | 7 | 0.00078 (0.00010) | 0.00082 (0.00012) | 2.48 | 0.038 | 0.086 |
|  | 9 | 0.00085 (0.00014) | 0.00091 (0.00014) | 2.36 | 0.046 | 0.086 |
| Dorsolateral prefrontal cortex L  (BA 9) | 4 | 0.00080 (0.00003) | 0.00082 (0.00004) | 2.45 | 0.040 | 0.060 |
|  | 5 | 0.00080 (0.00003) | 0.00082 (0.00005) | 2.45 | 0.040 | 0.060 |
|  | 6 | 0.00080 (0.00005) | 0.00082 (0.00007) | 2.46 | 0.040 | 0.060 |
|  | 7 | 0.00079 (0.00006) | 0.00083 (0.00008) | 2.81 | 0.023 | 0.060 |
|  | 8 | 0.00081 (0.00009) | 0.00085 (0.00011) | 3.24 | 0.012 | 0.060 |
|  | 9 | 0.00086 (0.00010) | 0.00091 (0.00012) | 2.89 | 0.020 | 0.060 |
|  | 10 | 0.00114 (0.00013) | 0.00119 (0.00013) | 2.32 | 0.049 | 0.063 |
| Dorsolateral prefrontal cortex R  (BA 9) | 8 | 0.00078 (0.00008) | 0.00082 (0.00012) | 2.73 | 0.026 | 0.086 |
|  | 9 | 0.00083 (0.00011) | 0.00087 (0.00012) | 2.85 | 0.021 | 0.086 |
|  | 10 | 0.00109 (0.00012) | 0.00113 (0.00011) | 2.66 | 0.029 | 0.086 |
| Anterior prefrontal cortex L  (BA 10) | 10 | 0.00107 (0.00009) | 0.00111 (0.00008) | 2.51 | 0.037 | 0.293 |
| Anterior prefrontal cortex R  (BA 10) | 9 | 0.00079 (0.00005) | 0.00081 (0.00006) | 3.70 | 0.006 | 0.048* |
|  | 10 | 0.00099 (0.00009) | 0.00104 (0.00010) | 3.11 | 0.014 | 0.058 |
| Dorsolateral prefrontal cortex L  (BA 46) | 4 | 0.00081 (0.00003) | 0.00082 (0.00003) | 2.38 | 0.045 | 0.112 |
|  | 5 | 0.00081 (0.00003) | 0.00082 (0.00003) | 2.46 | 0.039 | 0.112 |
|  | 8 | 0.00081 (0.00004) | 0.00083 (0.00004) | 2.41 | 0.042 | 0.112 |

Abbreviations: L-left hemisphere, R-right hemisphere, BA-Brodmann’s area, rTMS-repetitive transcranial magnetic stimulation, SD-Standard Deviation, FDR-false discovery rate.

* indicates FDR corrected p value < 0.05

Table S3. The decreased mean diffusion values after rTMS excitation.

| **Gray matter regions** | **Laminar layers** | **Baseline** | **After rTMS excitation** | **t** | **p values** | |
| --- | --- | --- | --- | --- | --- | --- |
|  |  | **Mean (SD)** | |  | **uncorrected** | **FDR corrected** |
| Orbitofrontal area R  (BA 11) | 2 | 0.00074 (0.00003) | 0.00072 (0.00004) | -2.87 | 0.017 | 0.038* |
|  | 3 | 0.00077 (0.00003) | 0.00074 (0.00003) | -3.74 | 0.004 | 0.035* |
|  | 4 | 0.00077 (0.00004) | 0.00074 (0.00004) | -3.15 | 0.010 | 0.038* |
|  | 5 | 0.00077 (0.00005) | 0.00074 (0.00004) | -3.04 | 0.013 | 0.038* |
| Primary visual cortex (V1) L  (BA 17) | 10 | 0.00117 (0.00008) | 0.00114 (0.00007) | -2.42 | 0.036 | 0.237 |
| Ectosplenial area R  (BA 26) | 4 | 0.00080 (0.00005) | 0.00077 (0.00004) | -3.37 | 0.007 | 0.052 |
|  | 5 | 0.00081 (0.00004) | 0.00078 (0.00005) | -2.35 | 0.040 | 0.121 |
|  | 6 | 0.00084 (0.00004) | 0.00081 (0.00005) | -3.09 | 0.012 | 0.052 |
| Part of cingulate cortex L  (BA 30) | 9 | 0.00091 (0.00007) | 0.00087 (0.00008) | -3.44 | 0.006 | 0.057 |

Abbreviations: L-left hemisphere, R-right hemisphere, BA-Brodmann’s area, rTMS-repetitive transcranial magnetic stimulation, SD-Standard Deviation, FDR-false discovery rate.

* indicates FDR corrected p value < 0.05

Table S4. The increased mean diffusion values after rTMS excitation.

| **Gray matter regions** | **Laminar layers** | **Baseline** | **After rTMS excitation** | **t** | **p values** | |
| --- | --- | --- | --- | --- | --- | --- |
|  |  | **Mean (SD)** | |  | **uncorrected** | **FDR corrected** |
| Inferior temporal gyrus R  (BA 20) | 10 | 0.00090 (0.00006) | 0.00092 (0.00006) | 2.31 | 0.044 | 0.395 |
| Part of cingulate cortex R  (BA 30) | 10 | 0.00117 (0.00011) | 0.00123 (0.00013) | 2.32 | 0.043 | 0.383 |
| Dorsal entorhinal cortex R  (BA 34) | 5 | 0.00071 (0.00024) | 0.00074 (0.00025) | 2.76 | 0.020 | 0.164 |
|  | 8 | 0.00074 (0.00026) | 0.00079 (0.00027) | 2.28 | 0.046 | 0.164 |

Abbreviations: L-left hemisphere, R-right hemisphere, BA-Brodmann’s area, rTMS-repetitive transcranial magnetic stimulation, SD-Standard Deviation, FDR-false discovery rate.

Table S5. The differences in change values of fractional anisotropy after inhibitory and excitatory rTMS.

| **Gray matter regions** | **Laminar layers** | **△FA values after rTMS inhibition** | **△FA values after rTMS excitation** | **t** | **p value** | |
| --- | --- | --- | --- | --- | --- | --- |
|  |  | **Mean(SD)** | |  | **uncorrected** | **FDR corrected** |
| Primary Somatosensory Cortex1 R (BA1) | 4 | 0.017 (0.039) | -0.017 (0.018) | -2.57 | 0.019 | 0.087 |
|  | 5 | 0.014 (0.020) | -0.025 (0.029) | -3.42 | 0.003 | 0.027* |
| Primary Somatosensory Cortex3 L (BA3) | 2 | 0.006 (0.024) | -0.019 (0.027) | -2.20 | 0.041 | 0.370 |
| Ventral posterior cingulate cortex R (BA23) | 2 | 0.025 (0.023) | -0.001 (0.019) | -2.76 | 0.013 | 0.117 |
| Ventral anterior cingulate cortex. L (BA24) | 3 | 0.002 (0.012) | -0.016 (0.020) | -2.37 | 0.029 | 0.090 |
|  | 4 | 0.002 (0.016) | -0.019 (0.022) | -2.35 | 0.030 | 0.090 |
|  | 5 | 0.002 (0.013) | -0.015 (0.018) | -2.43 | 0.026 | 0.090 |
| Ventral anterior cingulate cortex. R (BA24) | 9 | 0.008 (0.011) | -0.010 (0.016) | -2.74 | 0.014 | 0.122 |
| Subgenual area R  (BA25) | 3 | 0.017 (0.012) | -0.010 (0.0320) | -2.37 | 0.029 | 0.075 |
|  | 4 | 0.021 (0.019) | -0.013 (0.032) | -2.85 | 0.011 | 0.048* |
|  | 5 | 0.023 (0.016) | -0.015 (0.028) | -3.67 | 0.002 | 0.016* |
|  | 6 | 0.019 (0.019) | -0.005 (0.025) | -2.31 | 0.033 | 0.075 |
| Part of cingulate cortex L  (BA30) | 2 | 0.010 (0.034) | -0.025 (0.036) | -2.25 | 0.037 | 0.167 |
|  | 3 | 0.009 (0.012) | -0.018 (0.033) | -2.31 | 0.033 | 0.167 |
| Part of cingulate cortex R  (BA30) | 2 | 0.009 (0.015) | -0.011 (0.022) | -2.36 | 0.029 | 0.073 |
|  | 3 | 0.009 (0.018) | -0.010 (0.014) | -2.65 | 0.016 | 0.073 |
|  | 4 | 0.008 (0.016) | -0.007 (0.013) | -2.25 | 0.037 | 0.073 |
|  | 6 | 0.006 (0.018) | -0.009 (0.011) | -2.20 | 0.041 | 0.073 |
|  | 8 | 0.013 (0.014) | -0.005 (0.019) | -2.32 | 0.032 | 0.073 |
| Dorsal anterior cingulate cortex L (BA32) | 4 | 0.006 (0.013) | -0.011 (0.021) | -2.12 | 0.048 | 0.188 |
| Dorsal anterior cingulate cortex R (BA32) | 7 | 0.005 (0.012) | -0.010 (0.017) | -2.18 | 0.042 | 0.208 |
| Temporal pole L  (BA38) | 8 | 0.014 (0.016) | -0.007 (0.021) | -2.39 | 0.028 | 0.143 |
| Pars orbitalis L  (BA47) | 2 | 0.011 (0.024) | -0.015 (0.023) | -2.45 | 0.025 | 0.048* |
|  | 6 | 0.004 (0.013) | -0.012 (0.016) | -2.43 | 0.026 | 0.048* |
|  | 7 | 0.007 (0.017) | -0.014 (0.013) | -3.10 | 0.006 | 0.028* |
|  | 8 | 0.005 (0.017) | -0.016 (0.013) | -3.23 | 0.005 | 0.028* |
|  | 9 | 0.003 (0.017) | -0.014 (0.014) | -2.42 | 0.026 | 0.048* |

Abbreviations: L-left hemisphere, R-right hemisphere, BA-Brodmann’s area, rTMS-repetitive transcranial magnetic stimulation, SD-Standard Deviation, △FA values (subgroup1)-fractional anisotropy values after inhibitiory/exicitatory stimulation minus fractional anisotropy values at baseline, FDR-false discovery rate.

* indicates FDR corrected p value < 0.05

Table S6. The differences in change values of mean diffusion after inhibitory and excitatory rTMS.

| **Gray matter regions** | **Laminar layers** | **△MD values after rTMS inhibition** | **△MD values after rTMS excitation** | **t** | **p value** | |
| --- | --- | --- | --- | --- | --- | --- |
|  |  | **Mean (SD)** | |  | **uncorrected** | **FDR corrected** |
| Primary Somatosensory Cortex R (BA1) | 4 | -0.000039 (0.000051) | 0.000004 (0.000036) | 2.19 | 0.042 | 0.188 |
|  | 5 | -0.000042 (0.000047) | 0.000009 (0.000043) | 2.49 | 0.023 | 0.188 |
| Primary Somatosensory Cortex R (BA3) | 9 | 0.000019 (0.000039) | -0.000019 (0.000032) | -2.38 | 0.028 | 0.250 |
| Anterior prefrontal cortex R (BA10) | 8 | 0.000021 (0.000028) | -0.000019 (0.000048) | -2.19 | 0.042 | 0.126 |
|  | 9 | 0.000028 (0.000023) | -0.000020 (0.000062) | -2.23 | 0.039 | 0.126 |
|  | 10 | 0.000052 (0.000050) | -0.000014 (0.000068) | -2.42 | 0.026 | 0.126 |
| Orbitofrontal area R (BA11) | 3 | 0.000004 (0.000028) | -0.000030 (0.000027) | -2.81 | 0.012 | 0.104 |
|  | 4 | 0.000004 (0.000034) | -0.000033 (0.000034) | -2.41 | 0.027 | 0.114 |
|  | 5 | 0.000002 (0.000038) | -0.000035 (0.000038) | -2.15 | 0.046 | 0.114 |
| Primary visual cortex L (BA17) | 10 | 0.000017 (0.000049) | -0.000032 (0.000141) | -2.37 | 0.029 | 0.231 |
| Ectosplenial area (BA26) | 4 | 0.000011 (0.000050) | -0.000031 (0.000031) | -2.30 | 0.034 | 0.215 |
|  | 5 | 0.000018 (0.000053) | -0.000024 (0.000034) | -2.12 | 0.048 | 0.215 |
| Part of cingulate cortex L (BA30) | 8 | 0.000024 (0.000046) | -0.000020 (0.000036) | -2.41 | 0.027 | 0.121 |
|  | 9 | 0.000026 (0.000054) | -0.000037 (0.000036) | -3.11 | 0.006 | 0.055 |
| Fusiform gyrus L (BA37) | 10 | 0.000014 (0.000028) | -0.000031 (0.000058) | -2.15 | 0.045 | 0.406 |
| Temporal pole L (BA38) | 5 | -0.000021 (0.000033) | 0.000011 (0.000030) | 2.33 | 0.032 | 0.198 |

Abbreviations: L-left hemisphere, R-right hemisphere, BA-Brodmann’s area, rTMS-repetitive transcranial magnetic stimulation, SD-Standard Deviation, △MD values=mean diffusion values after inhibitory/excitatory stimulation minus mean diffusion values at baseline, FDR-false discovery rate.

Figure S1. Example slices of evenly-distributed layers based on the equal-distance method of surface-based laminar analysis displaying on three orthogonal planes. (A) axial view; (B) coronal view; (C) sagittal view. The multiple layers are indicated by different colors.


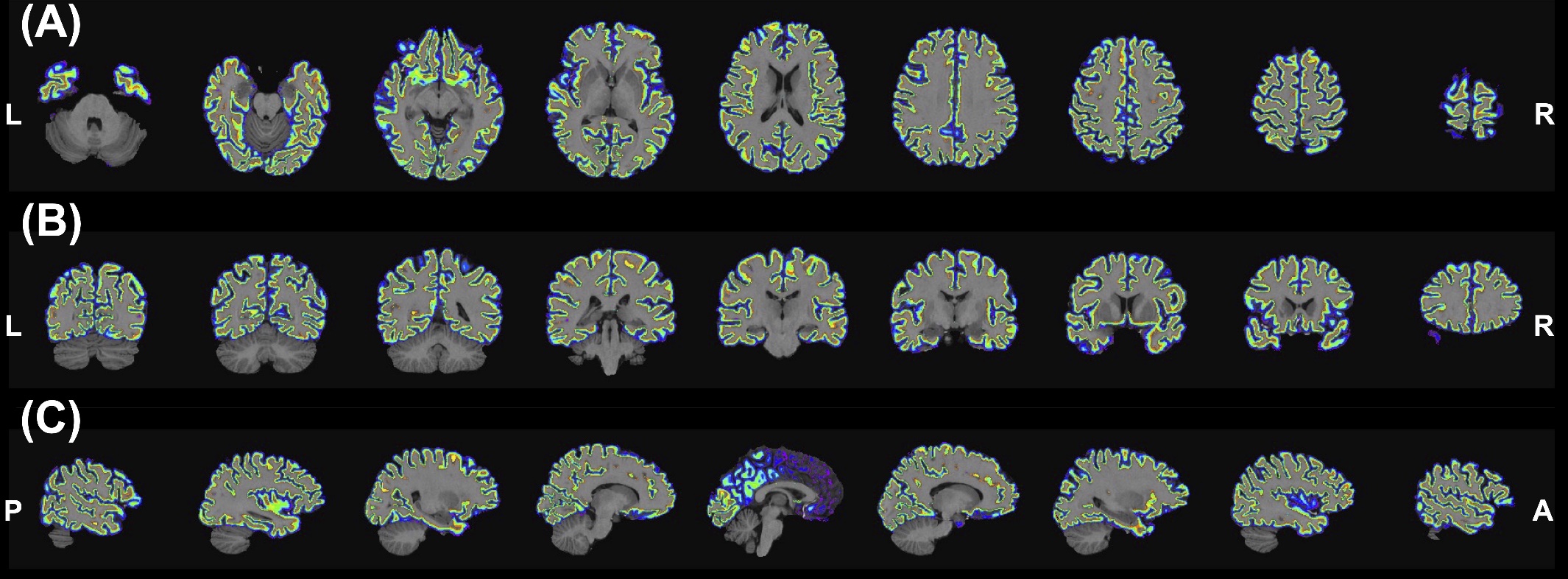


Abbreviations: L-left hemisphere, R-right hemisphere, P-posterior, A-anterior.
